# Optimizing Connectivity-Driven Brain Parcellation Using Ensemble Clustering

**DOI:** 10.1089/brain.2019.0722

**Published:** 2020-05-14

**Authors:** Anvar Kurmukov, Ayagoz Mussabaeva, Yulia Denisova, Daniel Moyer, Neda Jahanshad, Paul M. Thompson, Boris A. Gutman

**Affiliations:** ^1^ Institute for Information Transmission Problems, Russian Academy of Sciences, Moscow, Russia.; ^2^ Higher School of Economics, Moscow, Russia.; ^3^ Department of Biomedical Engineering, Medical Imaging Research Center, Illinois Institute of Technology, Chicago, Illinois, USA.; ^4^ Computer Science and Artificial Intelligence Laboratory (CSAIL), Massachusetts Institute of Technology, Cambridge, Massachusetts, USA.; ^5^ Imaging Genetics Center, Stevens Neuroimaging and Informatics Institute, Keck School of Medicine of USC, University of Southern California, Marina del Rey, California, USA.

**Keywords:** brain atlas, connectivity-based parcellation, diffusion MRI, ensemble clustering, human connectome, structural brain connectivity

## Abstract

This work addresses the problem of constructing a unified, topologically optimal connectivity-based brain atlas. The proposed approach aggregates an ensemble partition from individual parcellations without label agreement, providing a balance between sufficiently flexible individual parcellations and intuitive representation of the average topological structure of the connectome. The methods exploit a previously proposed dense connectivity representation, first performing graph-based hierarchical parcellation of individual brains, and subsequently aggregating the individual parcellations into a consensus parcellation. The search for consensus—based on the hard ensemble (HE) algorithm—approximately minimizes the sum of cluster membership distances, effectively estimating a pseudo-Karcher mean of individual parcellations. Computational stability, graph structure preservation, and biological relevance of the simplified representation resulting from the proposed parcellation are assessed on the Human Connectome Project data set. These aspects are assessed using (1) edge weight distribution divergence with respect to the dense connectome representation, (2) interhemispheric symmetry, (3) network characteristics' stability and agreement with respect to individually and anatomically parcellated networks, and (4) performance of the simplified connectome in a biological sex classification task. Ensemble parcellation was found to be highly stable with respect to subject sampling, outperforming anatomical atlases and other connectome-based parcellations in classification as well as preserving global connectome properties. The HE-based parcellation also showed a degree of symmetry comparable with anatomical atlases and a high degree of spatial contiguity without using explicit priors.

## Introduction

The ability to quantify how the human brain is interconnected *in vivo* has opened the door to a number of possible analyses. Connectome markers ranging from the simple graph descriptors such as edge weights and nodal degrees to sophisticated graph theoretical measures have all been invoked in the study of the brain. At the time of this writing, dozens of studies examining the effects of genetics and disease on structural and functional brain connectivity have been published (Horovitz and Horwitz, 2012; Jahanshad et al., 2013; Jiang et al., 2019; Lynall et al., 2010; Shah et al., 2017; Sun et al., 2014; Xu et al., 2016). In nearly all of these, brain parcellation plays a crucial role. Variations in parcellation significantly impact connectome reproducibility, derived graph theoretical measures, and the relevance of connectome measures with respect to biological questions of interest (de Reus and van den Heuvel, 2013; Petrov et al., 2017). Global topological properties of individual connectome models can vary substantially depending on the parcellation or set of nodes used (de Reus and van den Heuvel, 2013). This remains true even in the absence of any other variation, for example, for an identical tractography reconstruction, or the same resting state functional magnetic resonance imaging (rs-fMRI) preprocessing and correlation approach.

In short, the utility and interpretability of *in vivo* connectome measures depend to a great extent on the parcellation. For this reason, in recent years, much attention has been given to both parcellation-free approaches and parcellations derived specifically to attain some desired property (Prasad et al., 2014) in the implied connectivity graph. This approach uses individual densely sampled connectomes to drive the parcellation directly, leading to a more compact, connectivity-aware set of brain regions and resulting graph, as done in, for example, Parisot et al. (2016a). A comprehensive review of parcellation methods and their effects on the derived connectome quality is given in Arslan et al. (2018).

Because individual connectivity data are at once very informative and highly redundant, there is a great flexibility in how parcellations can be derived from dense, high-resolution graphs such as those based on vertices of a cortical surface mesh. It is possible, for example, to derive (1) a unified population-based atlas, (2) individual-level parcellations with cross-subject label mapping, or (3) individual parcellations with no intersubject label correspondence. While the first approach is appealing for its simplicity and ease of interpretation, the second and third may enable the researcher to reveal some individual aspect of the connectome that is lost in the aggregate atlas.

In this work, we attempt to bridge these three approaches by first constructing maximally flexible hierarchical parcellations, and then finding a unifying set of labels and parcels to maximize individual agreement. Several early approaches in connectivity-based parcellation relied on significant anatomical assumptions by representing the connectome as a vertex by anatomical region matrix (Draganski et al., 2008), by constraining the new parcels to lie within existing anatomical boundaries, or both (Lefranc et al., 2016). While such approaches are reasonable and result in a lower computational burden, their use may obfuscate some low-level topological features of individual connectomes. In particular, our requirement for maximum flexibility implies a preference for an anatomy-free dense connectome representation.

**FIG. 1.**
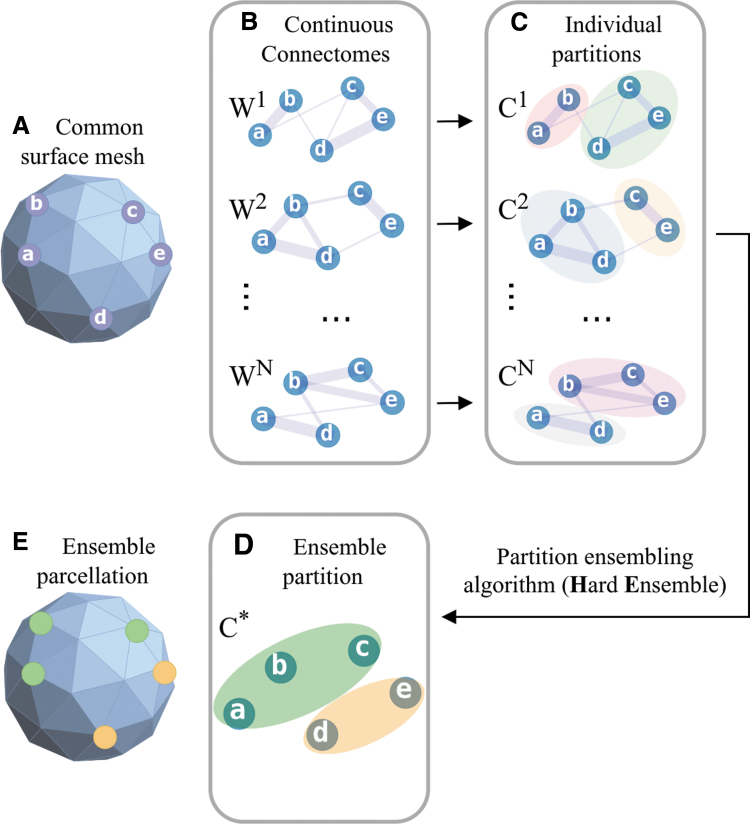
Overview of the proposed method. Individual continuous connectomes **(B)** are clustered independently **(C)**. Individual partitions are then ensembled, using the hard ensemble algorithm. The resulting ensemble partition **(D)** could be mapped onto the original mesh surface resulting in a unified parcellation **(E)**. Color images are available online.

Toward this end, we use a continuous representation of diffusion MRI (dMRI)-based brain connectivity (Moyer et al., 2017a) as our initial dense connectome. Continuous connectivity is a parcellation-free representation of tractography-based or “structural” connectomes that uses a generalization of the Poisson point process. Once individual parcellations are computed, we obtain a group-wise parcellation using a partition ensemble algorithm. The primary novelty of the approach comes from its ability to find an average partition without label correspondence in individual parcellations. Individual partitions retain the ability to compactly represent the unique topological structure of the connectome, while the average can be cast as a Karcher mean with respect to a measure of label agreement (Fig. 1).

All experiments are performed on 425 subjects from the Human Connectome Project (HCP). The ensemble construction procedure is stable and approximates the average parcellation well. Individual connectomes implied by the ensemble parcellation are shown to be more faithful and more compact representations of the underlying dense connectome than two popular anatomical atlases. We also compare our proposed method to the traditional means of constructing average partitions, namely by parcellating some aggregate graph structure constructed from individual graphs (Craddock et al., 2012; Parisot et al., 2016b). We use two realizations of this idea. In both cases, the ensemble partition shows better performance.

## Materials and Methods

In this section, we introduce methods for obtaining individual and group cortical parcellations from a set of *N* subjects. Throughout the article we work with two different entities, both represented as a network: brain surface mesh, denoted by *M*, and structural connectivity network, denoted by *W*. Individual brain surfaces are registered to the same reference (Glasser et al., 2013), and therefore, all subjects' surfaces share the same set of nodes $v_{1}\ldots v_{K}$. Individual connectivity network *W_i_* is represented as a K×K adjacency matrix constructed on the same set of nodes. Every edge $W_{u,v}^{i}$ represents how two cortical points *u* and *v* are connected in subject *i*. Thus, we have one-to-one correspondence between vertices of individual connectomes and the cortical surface mesh.

### Continuous connectome

The continuous connectome (ConCon) model treats each tract as an observation of an inhomogeneous symmetric Poisson point process with the intensity function given by$\lambda :\Omega \times \Omega \to {ℛ}^{+}\cup \left\{0\right\}$, where Ω denotes the union of two disjoint topologically spherical brain hemispheres representing cortical white matter boundaries. In practice, ConCon can be treated as a discrete connectivity graph with nodes defined by mesh vertices, that is, λ(x,y) is an analogue of an adjacency matrix. From this representation, a coarser discrete connectivity graph can be computed from any cortical parcellation *C*. We follow the definitions from Moyer et al. (2017a) and call $C={\left\{E_{u}\right\}}_{u=1,\ldots ,L}$ a parcellation of Ω if $E_{1},\ldots ,E_{L}\subseteq \Omega$ such that $\cup_{u=1}^{Z}E_{u}=\Omega$ and $E_{u}\cap E_{v}=0$ for u≠v, where *L* is the number of parcels. Edges between regions $E_{u},E_{v}$ can then be computed by the integration of the intensity function:

(1) $$\omega \left(E_{u},E_{v}\right)=\int_{E_{u}}\int_{E_{v}}\lambda \left(x,y\right)dxdy.$$

Due to properties of the Poisson process, $\omega \left(E_{u},E_{v}\right)$ is the expected number of observed tracts between *E_u_* and *E_v_*. In the context of connectomics, this is the expected edge strength.

### Individual parcellation

**FIG. 2.**
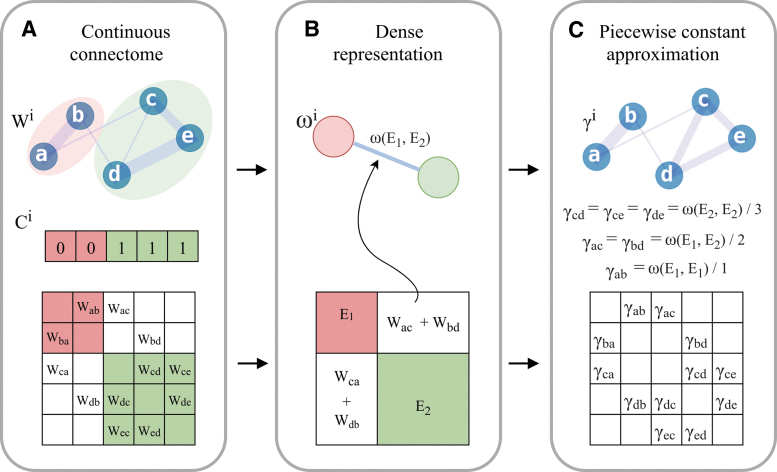
Piecewise constant connectome approximation. Individual ConCon s **(A)** are projected onto a “discrete” low-resolution representation **(B)**, which in turn is dilated into a piecewise constant approximation of the original continuous connectivity map (ConCon; **C**). ConCon continuous connectome. Color images are available online.

To obtain subject-level parcellation of *M*, we cluster vertices of the associated *W*. The resulting clustering *C* is represented by a vector of length $K:C=\left\{c_{1},\ldots ,c_{K}\right\}$, such that *c_j_* is the “color” or assigned cluster index of node *y_j_* of network *W*. We treat partitions that are different up to cluster index permutation as equivalent; for example, [1,1,1,0,0], [0,0,0,1,1], and [2,2,2,5,5] all represent the same partition of five objects. As the nodes of *W* and vertices of *M* are homologous across all subjects, we treat clusters of *W* as parcels of *M* (Fig. 1E, F). The process of applying a parcellation onto matrix *W* is represented in Figure 2A and B. Throughout the article, we use the terms “cluster,” “community,” “parcel,” and “color” (“node color”) interchangeably. We note that none of the individual or consensus connectome-based clustering approaches described below uses explicit contiguity priors. A cluster may contain several spatially disjoint groupings of mesh vertices.

Intuitively, graph clustering seeks to group graph nodes into clusters in such a way that nodes within the same cluster are densely connected, while intercluster connections are sparse. In this way, the original graph topology is preserved with the benefit of a more parsimonious representation. There are several ways to formalize this intuition. Here, we cluster subject-level connectomes by optimizing their graph modularity score, defined for a given clustering *C* as

(2) $$Q\left(W,C\right)=\frac{1}{m}\sum_{u,v}^{K}\left(W_{u,v}-\frac{d_{u}d_{v}}{m}\right)\delta \left(u,v\right),$$

where $d_{u}=\sum_{l}^{K}W_{u,v}$ is the degree of node *u*, and δ(u,v) is the Kronecker delta.

We use the Louvain modularity algorithm (Blondel et al., 2008), as it has shown good results in multiple neuroimaging studies (Kurmukov et al., 2017; Meunier et al., 2010; Nicolini et al., 2017; Taylor et al., 2017; Williams et al., 2019). We note that the overall goal of this work—approximating average parcellations—is agnostic with respect to the individual clustering algorithm. The Louvain approach initializes all nodes as separate clusters and applies an iterative two-step procedure. The first step performs a greedy modularity optimization by iteratively clustering nodes so long as the new membership assignment increases the modularity score. The second step builds a new metagraph, whose nodes are communities from the previous step, while the edges are defined as in Equation (1). The algorithm cycles over these steps iteratively, until further cluster merging ceases to increase modularity (Blondel et al., 2008).

**FIG. 8.**
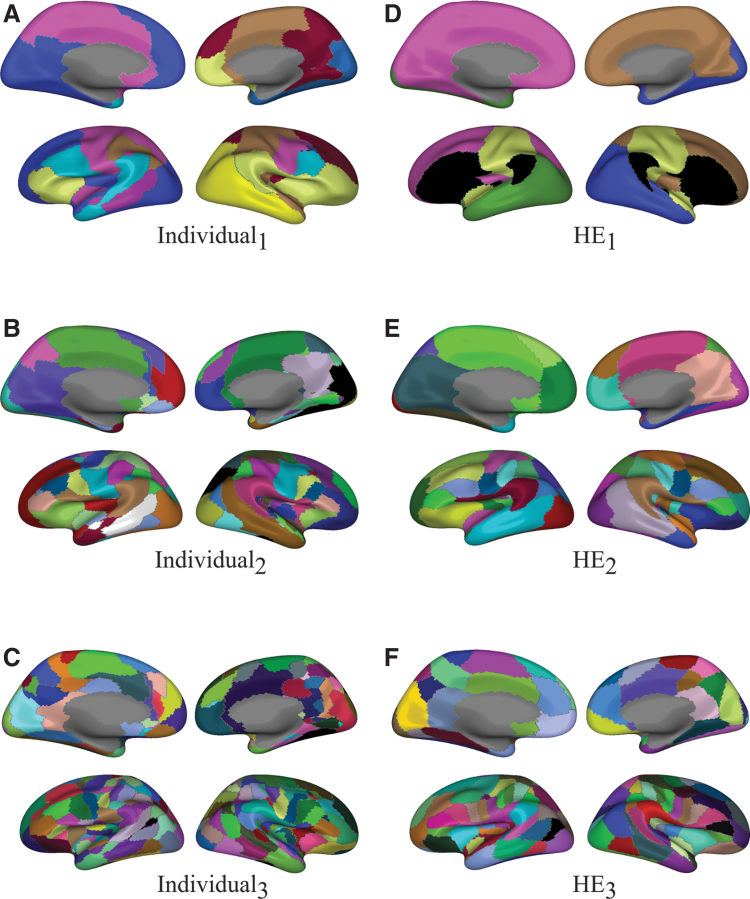
Hierarchical parcellation. Left: an individual parcellation; Right: group parcellation. All parcellations were derived from networks with 10% strongest edges. Note that regions of successive HE parcellations **(E–D, F–E,** and **F–D)** are not exactly hierarchically embedded, as they were obtained from the ensembling procedure. Subject-level parcellations are hierarchically embedded by construction **(B–A, C–B,** and **C–A)**. Color is random. Color images are available online.

The algorithm produces so-called hard clusters, or a partition: a set of disjoint communities, where every node is assigned to no more than one cluster. Since Louvain modularity tends to produce relatively large communities, we follow the hierarchical brain concept (Kurmukov et al., 2016; Meunier et al., 2010), repeating the clustering procedure recursively. After the initial parcellation, we further cluster each individual parcel as an independent graph. In this work, we repeat the process three times; Figure 8 shows the resulting individual parcellation for a sample subject, at all three levels. For each dense “continuous” connectome *W^i^*, this procedure yields a three-level hierarchically embedded partition: $C^{I},C^{II},C^{III}$. To prevent individual regions from becoming too small, we forbid subdivision of parcels containing fewer than 1% of all vertices.

We obtain partitions, and their corresponding connectome models [Eq. (1)], for every subject individually and independently at each refinement level. The number of nodes in the new connectome model corresponds to the number of communities in the individual parcellation *C*. In general, different subjects have different numbers of nodes without node correspondence across the data set.

### Ensemble clustering

Anatomical parcellations of different brains by the same atlas differ only in geometry. In this case, region labels are homologous across subjects and the notion of an average anatomical parcellation is equivalent to a registration problem. However, as we saw in the previous section, this is not the case for connectivity-based parcellations. Region labels are arbitrary and simple averaging of subject-level cluster assignments for each vertex is not possible. To address this, we turn to the concept of ensemble clustering. The goal of ensemble clustering is to aggregate multiple partitions of the same or homologous set of objects, in our case the mesh vertices across a set of registered cortical surface models. Formally, we wish to find a unifying partition from multiple individual partitions $C^{1},\ldots ,C^{N}$ of a set of arbitrary objects $v_{1}\ldots v_{K}$. We define the average partition of all *C^i^* as the Karcher mean over some partition distance d(∗,∗):


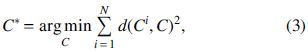


where $C^{*}$ denotes the desired average partition. Finding $C^{*}$ is generally NP-complete (Vega-Pons and Ruiz-Shulcloper, 2011), but several algorithms enable approximate solutions. Here, we use the hard ensemble (HE) approach (Dimitriadou et al., 2002). The HE algorithm is based on a greedy optimization of Equation (3). Partition distance d(∗,∗) is defined as the difference in membership functions up to permutation:


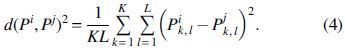


Here $P^{i},P^{j}$ are binary membership matrices of size K×L(L≤K) encoding two different data partitions, where *L* is the total number of communities in partition *C*:

(5) $$P_{k,l}=\left\{\begin{array}{c} 1ifC\left(k\right)=l\\ 0otherwise \end{array}\right..$$

For disjoint clusters, each row of *P* contains only one nonzero entry. As with the vectorial representation *C*, *P* is defined up to label index permutation, that is, up to any column permutation π. The optimization procedure is performed with respect to all possible π. In practice, as generally $L_{i}\neq L_{j}$, we set $L=max\left(L_{i},L_{J}\right)$ and pad the matrix with fewer clusters with all-zero columns.

HE combines multiple partitions $C_{i},\ldots ,C_{N}$ successively. First averaging a pair of subjects to obtain $C_{12}^{*}$, we proceed to find a weighted average $C_{123}^{*}$ of $C_{12}^{*}$ and subject *C*_3_, and so on until subject *C_N_* is averaged with $C_{1\ldots \left(N-1\right)}^{*}$ to obtain $C^{*}=C_{1\ldots N}^{*}$. The HE optimization procedure is order-dependent: averaging $C^{1},C^{2},C^{3}$ and $C^{2},C^{3},C^{1}$ may yield different results. We address this problem in the Experimental Pipeline section.

### Partitions based on aggregate graphs

To compare ensemble clustering to the standard methods of uniformly partitioning disparate graphs, we adapt two separate previously used approaches. The first is a simple average graph clustering. We compute the average ConCon: $W^{*}=\frac{1}{N}\sum_{i}W^{i}$, and cluster it using the Louvain approach over three hierarchical levels, just as we do for individual connectomes (Individual Parcellation section). We refer to this method as “Average.”

The second aggregate-based method is the cluster-based similarity partitioning algorithm (CSPA) (Strehl and Ghosh, 2002). CSPA defines the similarity between objects based on their co-occurrence in the same cluster across different partitions:

(6) $$S\left(v_{a},v_{b}\right)=\sum_{i=1}^{N}\delta \left(C^{i}\left(a\right),C^{i}\left(b\right)\right).$$

Here δ again is the Kronecker delta, and $C^{i}\left(a\right),C^{i}\left(b\right)$ represent colors of objects $v_{a},v_{b}$. Effectively, $S\left(v_{a},v_{b}\right)$ is the number of individual partitions that assign *v_a_* and *v_b_* to the same cluster. By clustering the graph *S*, CSPA obtains an approximate consensus partition.

Although several authors have used CSPA without mentioning it explicitly (Arslan et al., 2015; Craddock et al., 2012), their pipeline differs somewhat from ours, as CSPA, like HE, is also agnostic with respect to the clustering algorithm. For consistency, we again use the three-level Louvain modularity approach.

### Comparison metrics

In this section, we describe approaches to assess the quality of a unified parcellation. The first quality measure is the distance between the original continuous Poisson function λ(x,y) and its piecewise constant approximation, given by the following:

(7) $$\gamma \left(x,y\right)=\frac{1}{\left|E_{i}||E_{j}\right|}\omega \left(E_{i},E_{j}\right),$$

where $x\in E_{i}$ and $y\in E_{j}$, and $\left|E_{i}|,|E_{j}\right|$ are the expected nodal degrees of regions $E_{i},E_{j}$.

The natural way to compare two statistical distributions is to measure the distance between their probability density functions. Following Parisot et al. (2015), we use Kullback–Leibler (KL) divergence (Kullback and Leibler, 1951):

(8) $$KL\left(\lambda ,\gamma \right)=\int_{u\in \Omega \times \Omega }\lambda \left(u\right)log\frac{\lambda \left(u\right)}{\gamma \left(u\right)}du.$$

The intuition here is that networks derived using a “better” parcellation should have lower KL values with respect to the continuous representation, since they are better at capturing internal graph structure. Importantly, networks derived from parcellations with more parcels generally have lower KL at the cost of less compact representation.

To assess parcellation agreement objectively, that is, without using the metric in the Ensemble Clustering section, we use Adjusted Mutual Information (AMI) (Vinh et al., 2009), a normalized variant of Mutual Information (MI). AMI measures the similarity between two partitions, with the value of 1 corresponding to identical partitions and values close to zero corresponding to partitions that are very different. Given two different partitions *X* and *Y*, we build a K×K matrix *T* whose rows and columns correspond to clusters of *X* and *Y*, respectively. $T_{ij}=|X_{i}\cap Y_{j}|$, or the number of objects that are both in cluster *i* of partition *X* and in cluster *j* of partition *Y*. MI is then defined as usual:

(9) $$MI\left(X,Y\right)=\sum_{i}^{L_{X}}\sum_{j}^{L_{Y}}p_{ij}log\frac{p_{ij}}{p_{i}p_{j}},$$

where $L_{X},L_{Y}$ are the numbers of clusters in *X* and *Y*, $p_{i}=\frac{\left|X_{i}\right|}{K}$, and $p_{ij}=\frac{T_{ij}}{K}$. Among multiple options to adjust MI for chance, we use $AMI_{max}$:

(10) $$AMI_{max}\left(X,Y\right)=\frac{MI\left(X,Y\right)-E\left(MI\left(X,Y\right)\right)}{max\left(H\left(X\right),H\left(Y\right)\right)-E\left(MI\left(X,Y\right)\right)},$$

where $H\left(X\right)=-\sum_{i}^{Z_{X}}p_{i}logp_{i}$ is the entropy, and the expectation is computed based on the permutation model as in Vinh et al. (2009). MI and AMI are well suited for our purpose, as these measures are invariant to permutations of region indices just like our partition aggregation.

We use AMI in several contexts: to assess unified parcellation similarity to individual partitions, to compare different parcellations between themselves, and to assess HE ensembling stability with respect to the averaging order. Furthermore, as our left and right cortical meshes are in symmetric register, we use AMI to measure parcellation symmetry. We also introduce an exploratory measure of anatomical- and connectome-based parcellation agreement. We use the Sorensen–Dice score to measure overlap between regions of an anatomical atlas and the ensemble parcellation. For every anatomical atlas region *X_i_*, we compute its minimal cover of ensemble regions $Y^{*}={⋃}_{minY_{j}\in Y}Y_{j}∋X_{i}\subseteq Y^{*}$, that is, the minimal subset of regions *Y_j_* in parcellation *Y*, such that $X\subseteq Y^{*}.$ Parcellation agreement is then the Dice score between *X_i_* and $Y^{*}$:

(11) $$Dice=\frac{2|X_{i}\cap Y^{*}|}{\left|X_{i}|+|Y^{*}\right|}.$$

An additional potentially valuable property of a parcellation is the spatial contiguity of its resident parcels. Although it is a desirable property, unlike several previous approaches, we do not enforce it explicitly. To assess contiguity, we use the percentage of mesh vertices that are assigned to a spatially contiguous, simply connected parcel, that is, a label that defines exactly one piece of the cortex.

It has been observed that brain networks differ from various canonical networks of the same size mean nodal degree, for example, random, preferential attachment, or lattice networks, in specific ways (Bullmore and Sporns, 2009). It is known, for example, that connectomes are small-world networks, characterized by relatively high modularity and relatively short path lengths (Fornito et al., 2013). It then stands to reason that an appropriate unified parcellation will preserve these properties over some limited range of graph resolutions with respect to subject-optimized partitions. To test this, we take network properties derived from individual connectome-based partitions, and assess how well these are preserved when a unified partition is applied instead. An optimal partition should preserve these exactly, while a good partition should at least lead to a strong correlation between the ground truth values and consensus-based estimates. Here, we use two network characteristics: clustering coefficient (CC) and average path length (APL) (Rubinov and Sporns, 2010). Finally, we assess biological relevance of the simplified connectome representation using the receiver operating characteristic area under the curve (ROC AUC) score in a sex classification task.

### Data preprocessing

We analyzed data from 425 subjects: 167 men, mean age 28.0 (3.7), 258 women, mean age 29.3 (3.6) from the HCP S900 release (Van Essen et al., 2013). We reconstruct each subject's ConCon following the protocol in Moyer et al. (2017a). T1- (T1w) and diffusion-weighted images (DWI) were rigidly aligned to MNI 152 space. The HCP cohort has the following acquisition parameters: (1) T1w: flip angle: 8°; TI: 1000 msec; TE: 2.14 msec; TR: 2400 msec; voxel: 0.7 × 0.7 × 0.7 mm^3^; (2) DWI: flip angle: 78°; TE: 89.5 msec; TR: 5520 msec; 90 directions at each *b*-value; *b*-values: 1000, 2000, and 3000 sec/mm^2^; voxel size: 1.25 × 1.25 × 1.25 mm^3^.

All images were corrected for gradient nonuniformity. DWI was also corrected for motion and eddy current distortion. Cortical surface extraction, spherical registration, and labeling were performed using FreeSurfer version 5.3 recon-all (Fischl et al., 1999). All surfaces and labels were remeshed and resampled in the common spherical domain using a fifth-order icosahedral mesh (10,242 vertices) to construct meshes of reasonable resolution with dense vertex-to-vertex correspondence. Probabilistic streamline tractography was performed in 1.25 mm isotropic MNI 152 space using Dipy's implementation of constrained spherical deconvolution (Tournier et al., 2008) with a harmonic order of 8. We seeded tract streamlines at two random locations in each likely white matter voxel based on FSL FAST segmentation (Zhang et al., 2001). Streamline tracking followed random directions proportionally to the orientation distribution function at each step, starting bidirectionally from the seed. Only tracts longer than 5 mm with end-points in likely gray matter were retained. The ConCon construction was performed using kernel density estimation as per Moyer et al. (2017a), with a preset kernel parameter $\sigma =5\times 10^{-3}$.

### Experimental pipeline

It is generally established that all tractography reconstructions contain a substantial portion of false-positive tract models and connections, and this issue is commonly mitigated in practice by applying an arbitrary sparsity threshold (Thomas et al., 2014). To ensure that our method is robust with respect to network sparsity, we performed all experiments at 10 different network sparsity levels. We thresholded each ConCon representation to contain from 10% of the original edges (heaviest 10% edges) to 100% ( = no threshold) of the original density edges, in 10% increments.

In our experiments, we examine three important aspects of group parcellation. The first two are essential tests for any concept of averaging. The third aspect is related to connectomic analysis. In this context, applying a parcellation is a method to decrease dimensionality, and often to decrease noise, that is, reduce false-positive and false-negative edges (Zalesky et al., 2016) and thus increase the signal-to-noise ratio. In summary, an optimal parcellation should:

1. Be stable with respect to sample permutations or averaging random subsamples.
2. Be “in the middle” of the sample based on reasonable metrics.
3. Accurately approximate the structural connectivity of the brain.

We start by applying the three-level Louvain approach to ConCons. Next, we aggregate individual subject partitions and obtain consensus clustering using both CSPA and HE. We also compute the average connectome and cluster it using the Louvain algorithm. Finally, we apply Desikan–Killiany (DK) (Desikan et al., 2006) and Destrieux (Destrieux et al., 2010) parcellations. All network-based parcellation types are constructed from networks at each of the 10 sparsity levels. In total, for every ConCon representation, we obtain 110 low-resolution or “discrete” connectomes.

To measure intrinsic parcellation stability, we sample 100 random splits of HCP sample and compute an ensemble for each split. We compare all ensembles pairwise using AMI for a total of 100×99∕2=4950 comparisons. Finally, as the HE algorithm depends on averaging order, we randomly permute the subject order 100 times and proceeded as above with all pairwise comparisons.

We test the goodness-of-fit of our ensemble procedure based on two concepts:

1. Intrinsic label agreement.
2. Stability of global network characteristics (Rubinov and Sporns, 2010).

We take the mean AMI between a unified parcellation $C^{*}$ and all individual partitions, $\frac{1}{N}\sum_{i}^{N}AMI\left(C^{i},C^{*}\right)$, as a measure of “ensemble goodness.” We compare global network characteristics for connectomes derived using subject-level parcellations and ensemble parcellation. To assess network property preservation, we use the KL divergence on edge weight distributions. For sex classification, we use logistic regression with *l*_1_ penalty, using edge weights as features. To avoid overfitting, we derive an ensemble parcellation using one half of all subjects and apply it to the other half. Classification results are estimated on the second half of the data. We validate the results using a bilevel *k*-fold procedure: the first level is used to obtain the optimal Lasso parameter, and the second to assess classification performance.

Additional parcellation assessments include the following:

1. Interhemispheric symmetry in the ensemble parcellation, as our original cortical mesh representations are in symmetric vertex-wise register.
2. Spatial contiguity of the resulting group atlas.
3. Similarity between connectivity-driven parcellations (HE, CSPA, and Aver) and anatomical parcellations (DK and Destrieux).

All code was written in Python 3.7.3 and R 3.6.1, using the sklearn (Pedregosa et al., 2011), igraph (Csardi and Nepusz, 2006), and clue (Hornik, 2005) packages. All source code is available online https://github.com/kurmukovai/connectivity-brain-parcellation.

## Results

**FIG. 7.**
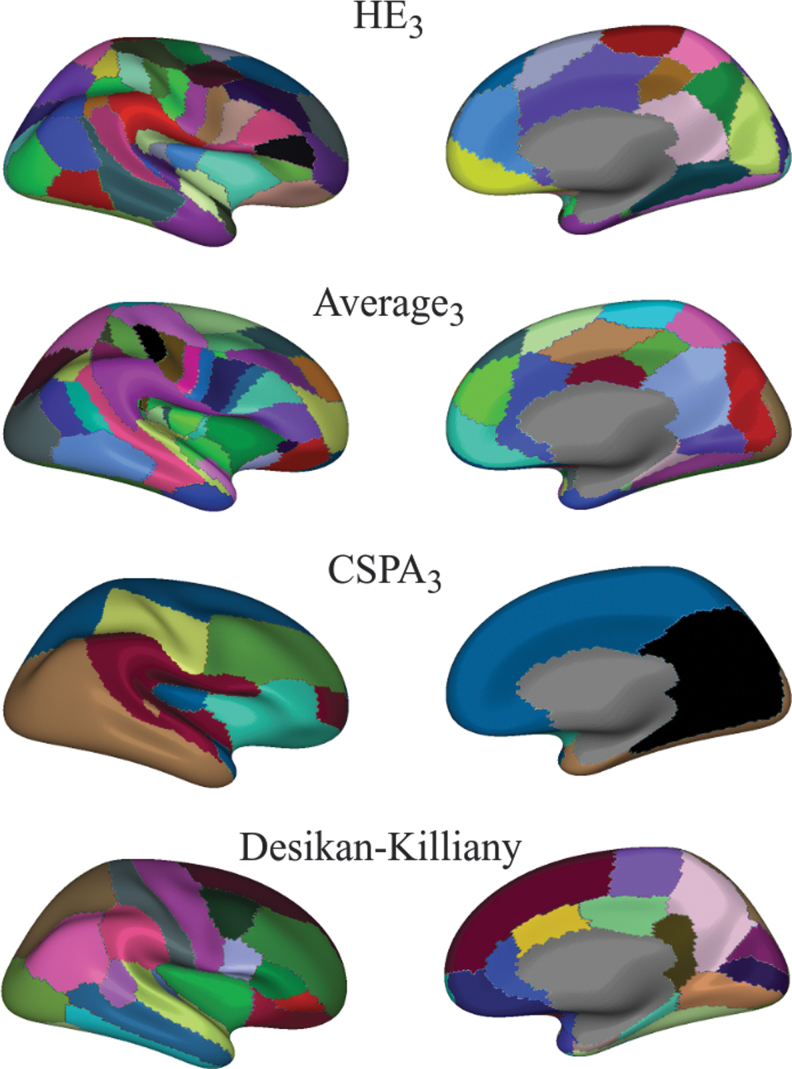
Group parcellation. All images correspond to the right hemisphere. HE, average and CSPA parcellations were derived from networks with 10% strongest edges. Color is random. CSPA, Cluster-based Similarity Partitioning Algorithm. Color images are available online.

Table 1 summarizes stability results with respect to edge thresholding (network sparsity) and subject sampling for all parcellations at level 3. The mean (std) AMI between HE parcellations with different subject order is 0.91 (0.02), implying a negligible effect of subject order. Average and HE approaches are stable in all cases, while CSPA is noticeably less stable with respect to subject sampling. CSPA, as implemented here, produces far “cruder” parcellations, that is, parcellations with substantially fewer regions, than the corresponding individual parcellations. The mean (std) number of regions in individual partitions was 9 (2), 35 (4), 96 (7) for $C^{I},C^{II},C^{III}$, respectively. CSPA produces seven regions for *C^I^*, 8 for $C^{II}$, and 10 for $C^{III}$. This can be partially explained by Louvain's tendency to produce few large parcels. We show all $C^{III}$ parcellations of 10%-sparse networks in Figure 7.

**Table 1.** Parcellation Stability with Respect to Different Metrics

Parcellation	No. of regions	Subject sampling similarity	Intramethod similarity	APL	Clustering coefficient
HE3	86 (2)	0.83 (0.3)	0.91(0.03)	1041.8 (0.1)	1029.0 (0.3)
Aver3	89 (3)	0.85 (0.07)	0.95 (0.05)	1041.7 (0.1)	1029.5 (0.3)
CSPA3	10 (1)	0.71 (0.08)	0.83 (0.02)	104178.6 (0.4)	10274.6 (7.0)
Individual3	96 (7)	(−)	0.83 (0.05)	1041.3 (0.2)	10210.1 (0.3)
Desikan	68 (−)	0.99 (0.00)	1.0 (−)	1043.3 (0.3)	1028.6 (0.3)
Destrieux	148 (−)	0.98 (0.00)	1.0 (−)	104.8 (0.1)	1029.1 (0.2)

Values in the table are mean and (standard deviation). For “number of clusters,” the std is measured over different edge thresholds; for “subject sampling,” std is measured over different samples; “intramethod similarity” represents average similarity between parcellations obtained using the same method, but different edge thresholds (e.g., HE3with 10%, 20%, …, 100% edges left), measured in terms of AMI. APL and CC are measured for a single threshold (10%), averaged over all subjects.

AMI, Adjusted Mutual Information; APL, average path length; CC, clustering coefficient.

**FIG. 4.**
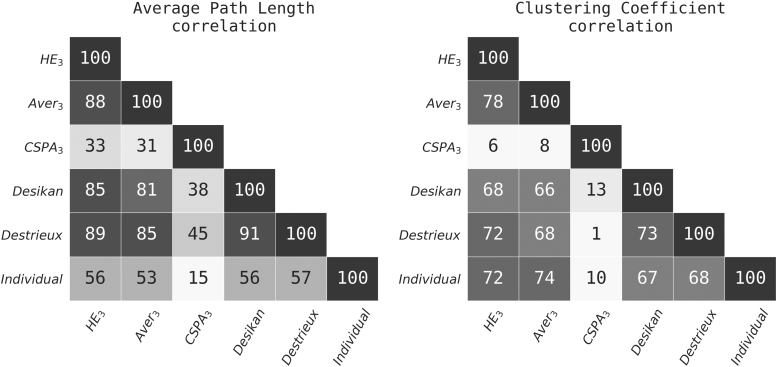
Pearson correlation ( × 100) between global network characteristics derived from different parcellations within the HCP sample. Left: Average path length; Right: Average clustering coefficient. “Individual” corresponds to level 3 individual subject parcellation. HCP, Human Connectome Project.

**FIG. 5.**
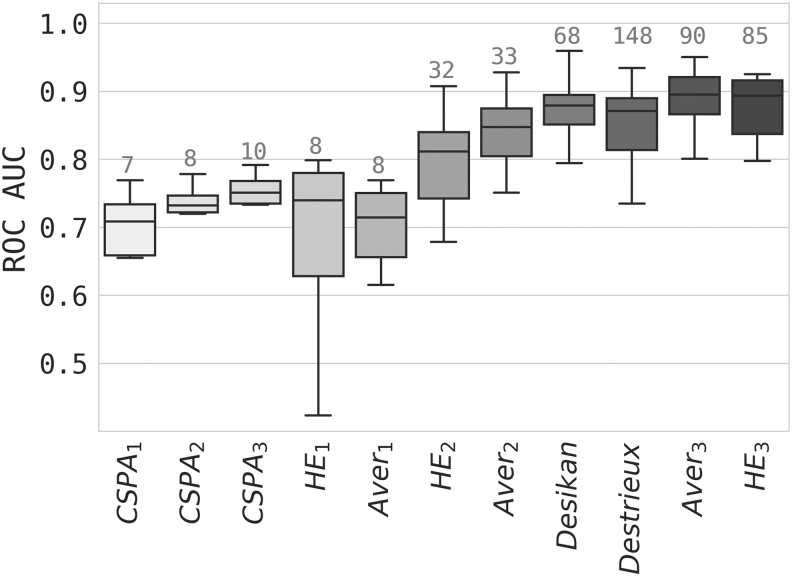
Classification performance in terms of ROC AUC (higher is better). Numbers above each box indicate the number of regions in the parcellation. Every box corresponds to a single method with the method-optimal sparsity threshold. Distribution is measured using fivefold cross-validation. ROC AUC, receiver operating characteristic area under the curve.

Mean AMI with respect to individual parcellations was highest for $HE_{3}$, followed closely by $Aver_{3}$ (Table 2). Table 1 shows mean network characteristics for all level 3 connectome-based and anatomical partitions used in this work. There is generally agreement within two standard deviations between anatomical atlas-based CC measures and connectome-derived ones, except for CSPA. Path length (APL) was also on the same order across methods with somewhat more disagreement, again with the exception of CSPA, which produced an order of magnitude larger estimates of both measures. At the subject level, there was strong correlation between CC/APL derived from the $HE_{3}$ partition and the anatomical as well as the individual connectome-based partitions. Figure 4 demonstrates correlations between individual, ensemble, and anatomical networks. It is noteworthy that $HE_{3}$ shows the highest correlation among the connectome-based aggregate partitions with both anatomical and individual networks for both measures, except for individual versus $Aver_{3}$APL correlation where it is very close. The ensemble partition appears to strike an optimal balance between anatomical and individual connectome-based parcellations. Biological sex classification is summarized in Figure 5. Surprisingly, even a parcellation with a small number of regions (HE_2_) performs nearly as well at this task as partitions with substantially more regions. $HE_{3}$ and $Aver_{3}$ show the best classification results, slightly outperforming the anatomical atlas networks.

**Table 2.** Parcellation Properties' Comparison

Parcellation	No. of clusters	Similarity with subject parcellation	Hemisphere symmetry	Spatial contiguity
HE1	8.4 (1)	0.53 (0.06)	0.25 (0.01)	0.93 (0.00)
HE2	31 (2)	0.65 (0.02)	0.57 (0.01)	0.94 (0.00)
HE3	86 (2)	**0.70 (0.01)**	**0.66 (0.01)**	**0.95 (0.00)**
Aver1	8 (1)	0.50 (0.05)	0.23 (0.00)	0.87 (0.08)
Aver2	33 (1)	0.64 (0.02)	0.52 (0.01)	0.89 (0.04)
Aver3	89 (3)	0.68 (0.01)	0.60 (0.01)	0.92 (0.01)
CSPA1	7 (1)	0.48 (0.06)	0.18 (0.03)	0.95 (0.02)
CSPA2	8 (1)	0.41 (0.02)	0.25 (0.01)	0.91 (0.06)
CSPA3	10 (1)	0.40 (0.01)	0.35 (0.02)	0.93 (0.05)

Results are averaged over all sparsity levels. Best and second-best result in every column is bold. Index denotes partition hierarchy level (e.g., HE2 is the parcellation derived using HE from individual partitions at level 2).

CSPA, cluster-based similarity partitioning algorithm; HE, hard ensemble.

**FIG. 6.**
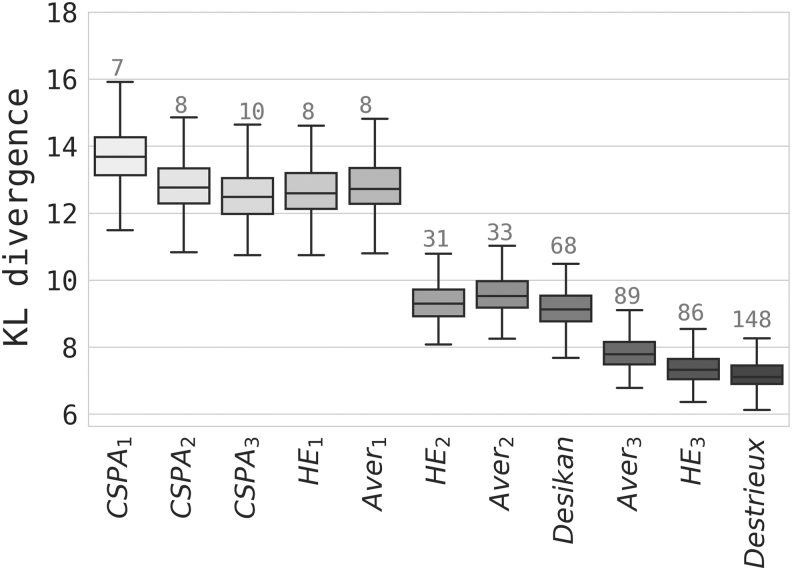
ConCon approximation goodness, in terms of KL divergence (lower is better). Numbers above each box indicate the number of regions in the parcellation. Each box corresponds to a whole HCP sample average for the given method at the 10% sparsity level. KL, Kullback–Leibler.

Results for piecewise-constant approximations of continuous connectivity based on cortical parcellations are shown in Figure 6. KL divergence is generally smaller for parcellations with more regions. However, our best parcellation, $HE_{3}$, has just over half of the number of regions in the Destrieux atlas, although the two are nearly equal in approximating dense networks. This holds true for $HE_{2}$ when compared with the DK parcellation. This result suggests that the intrinsic connectivity structure of the original ConCon representation is captured at least as well by the ensemble-based network, but with fewer parcels.

**FIG. 3.**
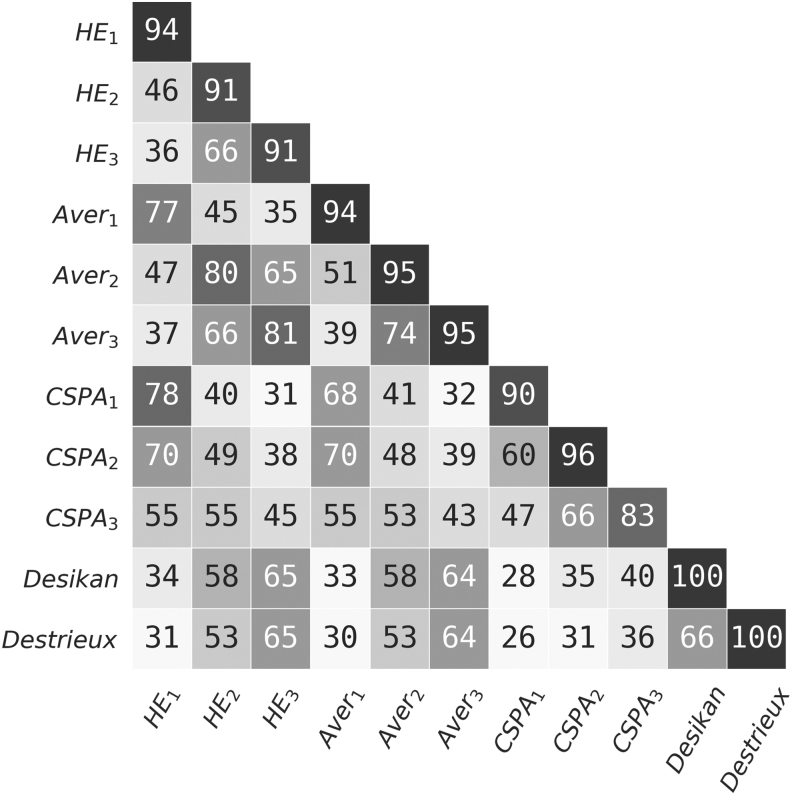
Similarity between different parcellations (AMI × 100). Values are averages across all sparsity levels. Diagonal values show average similarity between parcellations obtained using the same method, but different network sparsity levels. Note that anatomical parcellations do not depend on the individual connectome construction. AMI, Adjusted Mutual Information.

Finally, we analyze some natural parcellation properties. Table 2 shows that both HE and Average have high hemispheric symmetry and region contiguity. We also observe that the resulting connectivity parcellations are in many ways similar to anatomical parcellations, as shown in Figure 3. For example, the similarity between $HE_{3}$ and DK is about the same as the level of similarity between DK and Destrieux.

## Discussion

Recently, several approaches to construct connectome-based parcellations have been proposed. Central to many of these is some notion of graph clustering, with some enabling local or node-based group-level analysis based on a group partition, and others focusing on normalizing graph metrics without a unified parcellation. Variations on the first approach include clustering some aggregate graphs over corresponding nodes, including consensus clustering (Arslan and Rueckert, 2015; Arslan et al., 2015; Craddock et al., 2012), or using a multiview spectral clustering over several graphs simultaneously (Bickel and Scheffer, 2004; Parisot et al., 2016b).

The simplest of these methods clusters a connectome that comprised average edge weights in the sample. While easy to perform, this method leads to unified parcellations that ignore individual topological network differences (Lefranc et al., 2016). In some sense, this approach achieves high sensitivity but low specificity. Consensus clustering of aggregate graphs, such as CSPA, is another straightforward and well-studied approach. Here the issue is stability. As we have shown, consensus clustering relies on constructing a metagraph of cluster agreements. The results often vary substantially depending on the sample of connectomes. As with any optimization stability issue, well-defined priors, such as spatial constraints (Arslan and Rueckert, 2015), can partially alleviate this issue, but at the cost of imposing additional assumptions. With such methods, we often observe regions that are remarkably uniform in size and shape in contrast to known architectonic subdivisions of the cortex. An improvement on these approaches, multiview clustering allows one to find a clustering structure from multiple data sets simultaneously. This method is primarily developed for spectral clustering approaches and similarly lacks flexibility, for example, tending to find equally sized communities—a common issue with spectral clustering.

The approaches above share one additional shortcoming: the number of regions in a given parcellation must be predefined by the user. A desirable property for a group parcellation algorithm is the ability to optimize not only the composition but also the number of the regions automatically for network representation. An example of such an approach for individual connectome parcellation is given in Moyer et al. (2017b), where the authors exploit the ConCon Poisson process representation in a Bayesian nonparametric mixture model of connectivity. In this work, we instead sought a group parcellation method that addresses the issues above, automatically selects the number of regions, and preserves individual network properties.

Ensemble clustering offers a reasonable balance between these requirements. Using ensemble clustering in the context of brain parcellation is particularly interesting as it is agnostic to the choice of a specific clustering algorithm. To the best of our knowledge, this is the first application of ensemble clustering in this context. The Louvain + HE combination appears particularly potent, marrying the modularity maximization that is natural for cortical connectomes with a flexible ensemble partitioning that balances individual topology preservation and unified parcellation. It is worth stressing that the ensemble parcellation results in spatially contiguous parcels, without any specific constraints. Most prior work used spatial constraints to ensure this property, while Louvain + HE appear to derive reasonably contiguous regions solely from brain connectivity.

**FIG. 9.**
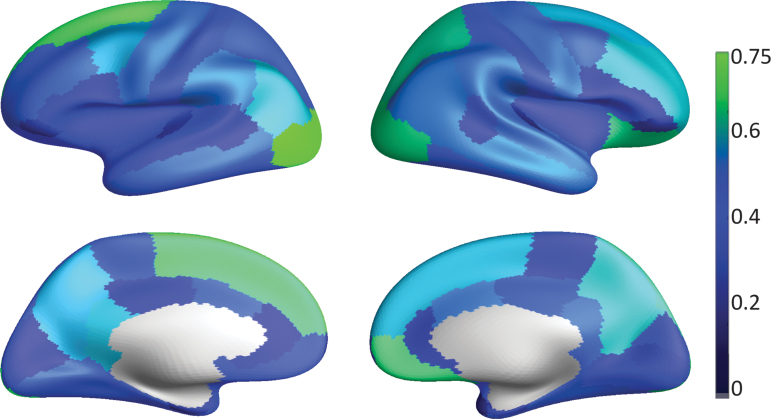
Overlap between the Desikan-Killiany atlas and the minimal ensemble parcellation covering. Anatomical regions in best agreement are the left lateral occipital sulcus (Dice = 0.83) and the left superior frontal gyrus (Dice = 0.73). Color images are available online.

As a final exploratory analysis, we compare minimal covering of the ensemble connectome parcels over their corresponding DK regions. The dice score between anatomical regions and their minimal connectomic coverings may be interpreted as follows: where the agreement is high, the anatomical regions capture well the modular decomposition of corticocortical connectivity. Conversely, where the agreement is low, cortical connectivity modules do not explain anatomical partitioning. A number of reasons can be postulated for regional differences in this measure. Here, we suggest two ideas. (1) Tractography quality varies with region (Thomas et al., 2014). (2) Areas dominated by corticocortical connections are more likely to agree with connectome modules. On the contrary, areas with increased connectivity to noncortical regions, for example, deep gray matter regions and the peripheral nervous system, are less likely to agree with parcels based only on connections to other parts of the cortex. Figure 9 appears to validate the second hypothesis: occipital and frontal areas have generally high agreement, while the sensory-motor strip and temporal areas have low agreement.

## Conclusion

We have presented an approach for generating unified connectivity-based human brain parcellations based on ensemble clustering. The method is based on finding a pseudo-Karcher mean over a set of individual partitions. Our approach outperforms standard anatomical parcellations based on several important metrics, including agreement with dense connectomes, improved relevance to biological questions, and improved symmetry. As our approach is entirely data-driven and requires no agreement between individual parcellation labels; it combines both the flexibility of individual parcellations and the interpretability of simple unified atlases. Experiments on independent groups show high reproducibility of the proposed parcellation, even though the ensembling procedure has several potential sources of uncertainty.

The analysis presented here is largely exploratory, and several questions remain open. Among these are robustness with respect to dMRI resolution and tractography type, stability with respect to different cohorts and individual parcellation types, and the effect of increasing sample size on the overall composition of the unified parcellation. Future exploration will address these questions, potentially developing a cohort-specific and cross-cohort meta-averaging procedure for large multisite brain connectivity studies.
